# Expression of the erythropoietin receptor by germline-derived cells - further support for a potential developmental link between the germline and hematopoiesis

**DOI:** 10.1186/1757-2215-7-66

**Published:** 2014-06-17

**Authors:** Malwina Suszynska, Agata Poniewierska-Baran, Pranesh Gunjal, Janina Ratajczak, Krzysztof Marycz, Sham S Kakar, Magda Kucia, Mariusz Z Ratajczak

**Affiliations:** 1 Stem Cell Institute at James Graham Brown Cancer Center, University of Louisville, 500 S. Floyd Street, Rm. 107, Louisville, KY 40202, USA; 2 Department of Physiology, Pomeranian Medical University, Szczecin, Poland; 3 University of Environmental and Life Sciences, Wroclaw, Poland

**Keywords:** EpoR, Germ line, Cancer development, VSELs, Ovarian cancer

## Abstract

**Background:**

Expressing several markers of migrating primordial germ cells (PGCs), the rare population of quiescent, bone marrow (BM)-residing very small embryonic-like stem cells (VSELs) can be specified like PGCs into hematopoietic stem/progenitor cells (HSPCs). These two properties of VSELs support the possibility of a developmental origin of HSPCs from migrating PGCs.

**Methods:**

To address a potential link between VSELs and germ line cells we analyzed by RT-PCR and FACS expression of erythropoietin receptor (EpoR) on murine bone marrow- and human umbilical cord blood-derived VSELs, murine and human teratocarcinoma cell lines and human ovarian cancer cells. A proper gating strategy and immunostaining excluded from FACS analysis potential contamination by erythroblasts. Furthermore, the transwell chemotaxis assays as well as adhesion and signaling studies were performed to demonstrate functionality of erythropoietin - EpoR axes on these cells.

**Results:**

We report here that murine and human VSELs as well as murine and human teratocarcinoma cell lines and ovarian cancer cell lines share a functional EpoR.

**Conclusions:**

Our data provide more evidence of a potential developmental link between germline cells, VSELs, and HSCs and sheds more light on the developmental hierarchy of the stem cell compartment in adult tissues.

## Introduction

The recent hot debate over the existence of developmentally primitive stem cells in bone marrow (BM) with broad differentiation potential has been fueled by the challenge these cells pose to the accepted hierarchy within the stem cell compartment of hematopoietic tissues [1]. Also surprising is the accumulating evidence that hematopoietic stem cells (HSCs) are specified from a population of migrating primordial germ cells (PGCs) during embryogenesis [2-4] and that stem cells with germ cell potential reside in adult BM [5,6].

In support of this intriguing linkage, HSCs and PGCs are highly migratory cells, and specification of the first primitive HSCs in the yolk sac blood islands as well as the origin of definitive HSCs in the aorta–gonado–mesonephros (AGM) region are chronologically and anatomically correlated with the developmental migration of PGCs in extra- and intra-embryonic tissues [7]. In addition, several papers have described the sharing of chromosomal aberrations between germline tumors and leukemias or lymphomas, which suggests their clonal origin [8-10]. Furthermore, it has been demonstrated in *in vitro* cultures that murine PGCs isolated from embryos, stem cells isolated from murine testes [4], and teratocarcinoma cell lines can be specified into hematopoietic stem/progenitor cells (HSPCs) [11].

Our recent work demonstrated the presence of small, quiescent, Sca-1^+^Lin^-^CD45^-^ stem cells in adult murine BM and small CD133^+^Lin^-^CD45^-^ cells in human BM and umbilical cord blood (UCB) [12-16]. These cells, under appropriate co-culture conditions with OP-9 stromal cells, can be specified into HSPCs [17], and, based on the presence of a primitive type of chromatin in their nuclei as well as expression of embryonic stem cell markers such as Oct-4 and Nanog, these small cells were named very small embryonic-like stem cells (VSELs). Interestingly, murine VSELs express several markers, such as *Stella, Prdm14, Fragilis, Blimp1, Nanos3,* and *Dnd1,* that are shared with migratory primordial germ cells PGCs [18,19]. Based on these observations, we have proposed a developmental link between PGCs, VSELs, and HSCs [17,19]. We have also hypothesized that these cells, if mutated, may give rise to certain types of malignancies [20], which may reconcile the presence of these cells in adult tissues with the more than 150-year-old embryonic rest hypothesis of cancer development.

The erythropoietin receptor (EpoR) is well known to be essential for production of red blood cells. Recently, however, evidence has accumulated that it is also expressed in non-hematopoietic tissues (e.g., some neuronal cells) [21]. Accordingly, EpoR has also been reported to be expressed by several types of malignant cells, including ovarian cancer cell lines [22,23]. These findings prompted us to evaluate the expression of EpoR on murine and human VSELs as well as murine and human germline-derived cell lines. The presence of EpoR on VSELs and germline cell lines provides new evidence for a developmental link between VSELs, HSCs, and the germline and sheds more light on the developmental hierarchy within the adult stem cell compartment.

## Materials and methods

### Murine BM and human umbilical cord blood cells and cell lines

Mononuclear cells were isolated from BM of 4–6-week-old C57Bl/6 mice and human umbilical cord blood in agreement with approval by the Animal Care and Use Committee (IACUC) and Institutional Review Board (IRB) of the University of Louisville (Louisville, KY). All cell lines employed in our studies (P19, NTERA2, A2780) were purchased from ATCC (Manassas, VA, USA). C57Bl/6 mice were purchased from Jackson Laboratories (Bar Harbor, ME, USA). Clinical-grade UCB research units were shipped from the Cleveland Cord Blood Center.

### Isolation of murine VSELs

Mice were sacrificed and bone marrow was flushed from the femur and tibia. A single-cell suspension was obtained by agitation through the syringe. Cells were lysed in BD lysing buffer (BD Pharmingen) for 10 min at room temperature and washed twice in RPMI medium with 2% FBS. The cell suspension was stained for the VSEL phenotype using antibodies: anti-CD45R/B220 (PE, clone RA-6B2), anti-Gr-1 (PE, clone RB6-8 C5), anti-T cell receptor αβ (PE, clone H57-5970), anti-T cell receptor ɣδ (PE, clone GL3), anti-CD11b (PE, clone M1/70), anti-Ter119 (PE, clone TER-119), anti-CD45 (allophycocyanin–Cy7, clone 30 F11) and anti-Ly-6A/E (also known as Sca-1, PE–Cy5 or Alexa Fluor 647, clone E13-161.7) for 30 minutes on ice. Cells were washed and resuspended in RPMI medium with 2% fetal bovine serum. Populations of Lin^-^/CD45^-^/Sca-1^+^ (VSELs) and Lin^-^/CD45^+^/Sca-1^+^ (HSCs) were sorted with a Moflo XDP cell sorter (Beckman Coulter).

### Isolation of human VSELs

Clinical-grade UCB research units were shipped from Cleveland Cord Blood Center. Total nucleated cells (TNCs) were isolated by hypotonic lysing of red blood cells (RBCs) employing BD PharmLyse lysing buffer (×2) for 10 minutes at room temperature. After a washing step, magnetic labeling of TNCs for the depletion of mature hematopoietic lineage-positive cells (Lineage Cell Depletion kit, Miltenyi Biotec) was performed. The population of lineage-negative cells was isolated by using an autoMACS separator (Miltenyi Biotec GMBH, Germany) and incubated with antibodies: anti-CD2 (FITC, clone RPA-2.10), anti-CD3 (FITC, clone UCHT1), anti-CD14 (FITC, clone M5E2), anti-CD16 (FITC, clone 3G8), anti-CD19 (FITC, clone HIB19), anti-CD24 (FITC, clone ML5), anti-CD56 (also known as N-CAM, FITC, clone NCAM16.2), anti-CD66b (FITC, clone G10F5), anti-CD235a (FITC, clone GA-R2 [HIR2]), anti-CD45 (PE or V450, clone HI30), and anti-CD34 (APC or PE, clone 581) or anti-CD133/1 (APC or PE, clone AC133). Cells were washed, resuspended, and sorted using a MoFlo XDP cell sorter (Beckman Coulter) to obtain populations enriched in VSELs (Lin^-^/CD45^-^/CD133^+^ or Lin^-^/CD45^-^/CD34^+^) as well as hematopoietic stem/progenitor cells (HSPCs, Lin^-^/CD45^+^/CD133^+^ or Lin^-^/CD45^+^/CD34^+^).

### In vivo BrdU labelling studies with murine VSELs

C57BL/6 mice (4 to 6 weeks old) were injected subcutaneously with human recombinant erythropoietin (Epogen, 25 U/day, 10 times, every third day) and injected intra-peritoneally with BrdU solution (1 mg/animal, BD Pharmingen) every working day. Control mice were injected with saline and BrdU solution. After 30 days, mice were sacrificed, and a single-cell suspension was obtained and stained for the VSEL phenotype as previously described. After cell-surface staining of cells, the FITC BrdU Flow kit (BD Pharmingen) staining protocol was used, which includes fixation and permeabilization of cells, treatment of cells with DNase to expose incorporated BrdU, and staining with anti-BrdU–FITC antibody. After washing, samples were analyzed using a BD LSR II flow cytometer (BD Biosciences). At least 10^6^ events were acquired and analyzed using BD FACSDiva software.

### Analysis of VSELs for the presence of erythroid markers

A single-cell suspension was obtained from 4- to 6-week-old C57BL/6 mice and stained for the VSEL phenotype as previously described. Additionally, cells ware stained for markers characteristic of erythroid cells using antibodies: anti-Ter119 (V450, clone TER-119) and anti-CD71 (FITC, clone C2) for 30 minutes on ice. After washing, cells were fixed and permeabilized with BD Cytofix/Cytoperm™ solution for 15 minutes on ice. The fluorescent DNA intercalator 7AAD was added to samples to visualize only flow cytometer events with DNA content. At least 2×10^6^ nucleated events were acquired using a BD LSR II flow cytometer (BD Biosciences, San Jose, CA).

### Detection of EpoR expression by FACS

Single-cell suspensions of mononuclear cells were obtained from UCB research units and stained for the VSEL phenotype as previously described. Additionally, cells ware stained for markers characteristic of erythroid cells using antibodies: anti-CD235a (PE–Cy7, clone GA-R2 [HIR2]) and anti-EpoR (APC, clone 38421) for 30 minutes on ice. After washing, cells were fixed and permeabilized with BD Cytofix/Cytoperm™ solution for 15 minutes on ice. 7AAD was added to samples to visualize only events exhibiting DNA content. At least 2×10^6^ nucleated events were acquired using a BD LSR II flow cytometer (BD Biosciences, San Jose, CA).

### Immunohistochemical staining of FACS-sorted mBM-derived VSELs

Lin^-^/CD45^-^/Sca-1^+^ murine VSELs as well as HSCs were sorted, plated on 22-mm diameter plates coated with poly-L-lysine (P9155, Sigma, MO, USA), and incubated for 24 h. Subsequently, cells were fixed in 3.5% paraformaldehyde for 15 min, permeabilized by 0.1% Triton X100, washed in PBS, and pre-blocked with 2.5% BSA for 2.5 h at RT to avoid nonspecific binding of antibodies. Immunocytochemistry for POU5F1 (clone 7 F9.2, mouse monoclonal IgG1κ, Millipore, MA, USA), and EpoR (rabbit polyclonal IgG, Abcam, Cambridge, MA, USA) proteins was performed with appropriate secondary Alexa Fluor 488 goat anti-mouse IgG and Alexa Fluor 594 goat anti-rabbit IgG antibodies. Nuclei were stained with DAPI (Invitrogen) for 20 min at 37°C. All images were captured with an Olympus FV1000 confocal microscope.

### Detection of EpoR expression by real-time quantitative PCR (RQ-PCR)

Total RNA from FACS-sorted or cultured cells was isolated using the RNeasy Mini kit (Qiagen Inc, Valencia, CA, USA), with removal of genomic DNA using the DNA-free™ kit (Life Technologies, Grand Island, NY, USA). cDNA was prepared with Taqman Reverse Transcription Reagents (Applied Biosystems, Grand Island, NY, USA ), according to the manufacturer’s instructions. Quantitative assessment of mRNA levels of target genes was performed by RQ-PCR using an ABI Prism 7500 sequence detection system (Applied Biosystems). The cDNA templates from each cell were amplified using SYBR Green PCR master mix (Applied Biosystems), and specific primers (*mEPO R:* forward, 5′-cattctggtcctcatctcgctg-3′, reverse, 5′-tgaagagaccctcaaactcgct-3′; *hEPO R:* forward, 5′-ccatggacactgtgccctg-3′, reverse, 5′- ccatcggataagccccctt-3′) were designed with Primer Express software (Applied Biosystems). The threshold cycle (Ct), the cycle number at which the fluorescence of the amplified gene reached a fixed threshold, was subsequently determined, and the relative quantification of the expression level of target genes was performed with the 2^–ΔΔCt^ method.

### Conventional RT-PCR

Total RNA from various cells was isolated using the RNeasy Mini kit (Qiagen Inc.), including treatment with DNase I (Qiagen Inc.). The mRNA (200 ng) was reverse-transcribed with Taqman Reverse Transcription Reagents (Applied Biosystems) according to the manufacturer’s instructions. The resulting cDNA fragments were amplified using Amplitaq Gold at 1 cycle of 8 min at 95°C, 2 cycles of 2 min at 95°C, 1 min at 60°C, 1 min at 72°C, and subsequently by 37 cycles of 30 s at 95°C, 1 min at 60°C, 1 min at 72°C, and 1 cycle of 10 min at 72°C by using sequence-specific primers (*mEpoR:* F5′-AGGGCTGCATCATGGACAAA-3′, R5′-GGTGATAGCGAGGAGAACCG-3′; *hEpoR:* F5′-CTCCCTTTGTCTCCTGCTCG-3′, R5′-TAGGCAGCGAACACCAGAAG-3′). All primers were designed using the NCBI/Primer-Blast program, as at least one primer included an exon–intron boundary.

### Chemotaxis assay

Chemotaxis assays were performed in a modified Boyden’s chamber with 8-μm pore polycarbonate membrane inserts (Costar Transwell; Corning Costar, Lowell, MA, USA) as described previously. In brief, cells detached with 0.25% trypsin were seeded into the upper chamber of an insert at a density of 6 × 10^4^ in 100 μl. The lower chamber was filled with pre-warmed culture medium containing test reagents. Medium supplemented with 0.5% BSA was used as a negative control. After 24 hours, the inserts were removed from the Transwell supports. The cells that had not migrated were scraped off with cotton swap from the upper membrane, and the cells that had transmigrated to the lower side of the membrane were fixed and stained with HEMA 3 (protocol, Fisher Scientific, Pittsburgh, PA) and counted on the lower side of the membrane using an inverted microscope.

### Cell adhesion assay

P19 murine teratocarcinoma cells were made quiescent by incubation for 3 h at 37°C in αMEM medium supplemented with 0.5% BSA. The cells were then stimulated with EpO 0.1, 1, and 100 U/ml for 5 min at 37°C, then added to plates treated with fibronectin (10 μg/ml) and incubated for 5 min at 37°C. After the non-adherent cells had been discarded, cells that adhered to the stromal cells were stained and counted under an inverted fluorescence microscope.

### Signaling studies

Human and murine teratocarcinoma or ovarian cancer cells were kept overnight in medium containing low levels of bovine serum albumin (BSA, 0.5%) to render the cells quiescent. After the cells were stimulated with EpO (1 or 100 U/ml for the P19 cell line; 0.5 or 10 U/ml for the NTERA2 cell line; 50, 100, or 150 U/ml for the A2780 cell line) for 5 min, cells were lysed for 20 min on ice in RIPA lysis buffer containing protease and phosphatase inhibitors (Santa Cruz Biotech, Santa Cruz, CA). The extracted proteins were separated on a 4–12% SDS-PAGE gel and transferred to a PVDF membrane. The phosphorylation of the p44/42 mitogen-activated kinase (phospho-p44/42 MAPK) was detected by phospho-specific p44/42 MAPK mouse and rabbit polyclonal antibodies (Cell Signaling, Danvers, MA, USA) with HRP-conjugated goat anti-mouse and anti-rabbit secondary antibodies (Santa Cruz Biotech). Equal loading of protein in all the lanes was evaluated by stripping the blots and reprobing with anti-p42/44 MAPK monoclonal antibody (clone no. 9102, Cell Signaling). The membranes were developed with an enhanced chemiluminescence (ECL) reagent (Amersham Life Sciences, Arlington Heights, IL), dried, and subsequently exposed to film (Hyperfilm, Amersham Life Sciences).

### Statistical analysis

All results were presented as mean ± SD. Statistical analysis of the data was performed using Student’s t-test for unpaired samples, with p < 0.05 considered significant.

## Results

### Effect of EpO on proliferation of VSELs in murine BM

**Figure 1:**
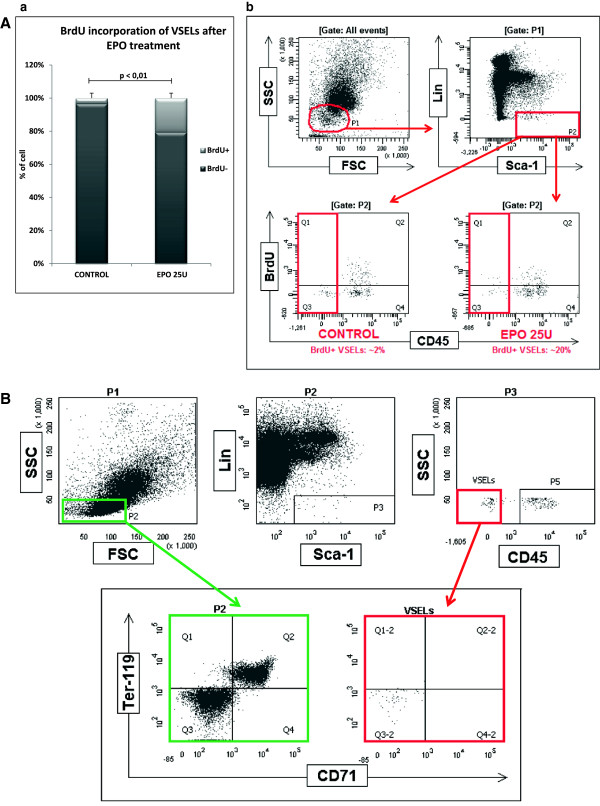
**Erythropoietin induces proliferation of quiescent BM VSELs. Panel A.** BrdU incorporation into VSELs after EPO treatment. **(a)** The percentage of VSELs that incorporated BrdU into newly synthesized DNA. After treatment with 10 doses of EPO at 25 U/dose, ~20% of VSELs are BrdU^+^, in contrast to the control group (~2% of VSELs are BrdU^+^). **(b)** Incorporation of BrdU was measured by FACS. Representative FACS analysis of six experiments is shown. **Panel B.** Analysis of VSELs for the presence of erythroid markers. Cells were fixed and stained with 7AAD to show only events exhibiting DNA content, gate P1 (not shown). Gate P2 includes small, agranular cells. Gate P3 includes Sca-1^+^/Lin^-^ cells, which are visualized on the next dot plot as CD45-negative cells (VSELs) and CD45-positive cells (HSPCs). Erythroblast markers CD 71 and Ter-119 are not expressed on VSELs (right, lower panel) in contrast to control cells, where some of the cells express CD71 and Ter-119 among the population from the P2 gate (left, lower panel). One representative dot plot analysis out of three is shown.

As previously described, VSELs are quiescent cells due to epigenetic changes in certain paternally imprinted genes [24]. Since the first hematopoietic cells that emerge during embryogenesis in yolk sac blood islands are primitive erythroid cells, we became interested in whether prolonged administration of EpO induces proliferation of these cells. To address this question, normal 6-week-old animals were injected s.c. with human recombinant EpO (25 U/day, 10 times, every third day) along with BrdU or saline (control animals). Ten days later, animals were sacrificed, and we analyzed the number of VSELs and HSCs in the cell cycle according to BrdU incorporation as detected by FACS (Figure 1 panel A). We observed that the number of BrdU VSELs in mice injected with BrdU increased from ~ 2% to ~20% compared with control saline-injected mice.Importantly, to exclude potential contamination of VSELs by the more differentiated erythroid progenitors, we analyzed murine BM-purified VSELs for expression of the erythroid cell markers Ter119 and CD71, and, as shown in Figure 1 panel B, murine BM VSELs do not express those antigens.

### Murine and human VSELs express EpoR

**Figure 2:**
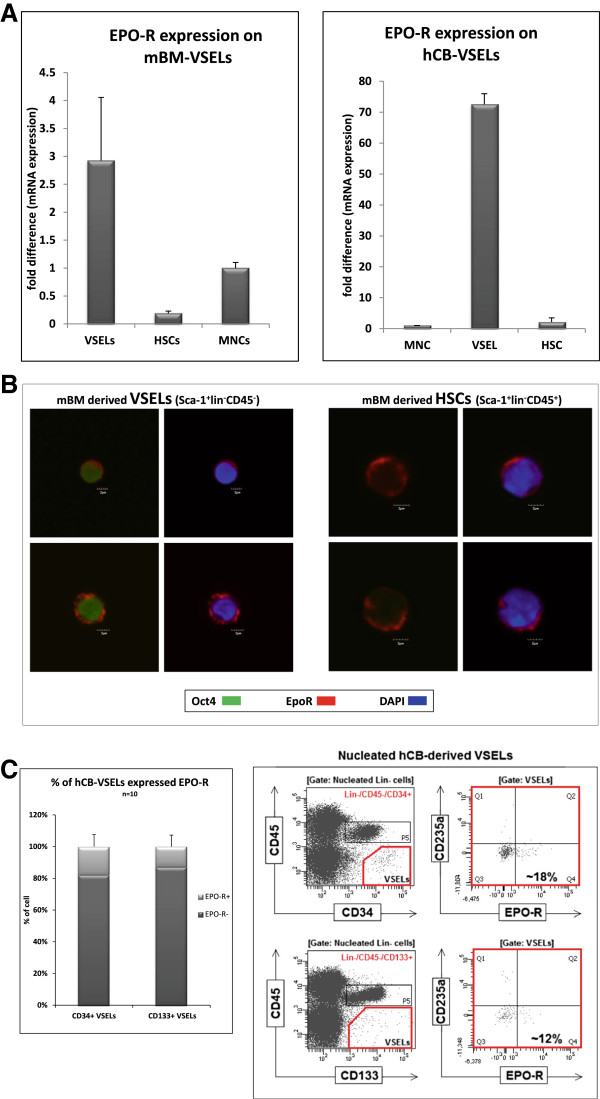
**EpoR is expressed by murine and human VSELs. Panel A.** RQ-PCR results for EpoR expression by purified murine (left panel) and human (right panel) VSELs, HSCs, and MNCs. Combined data from three independent experiments are shown. **Panel B**. Immunostaining of EpoR and Oct4 protein in purified murine VSELs (left panel) and HSCs (right panel). The images were acquired using an Olympus FV1000 confocal microscope. A representative staining is shown. The experiment was performed on three independent cell sorts. **Panel C.** Detection of EpoR expression by FACS among hCB-derived VSELs. The left panel presents the percentages of CD34^+^ and CD133^+^ VSELs that express EpoR. The level of expression of EpoR on UCB-derived VSELs is ~20% for the population of Lin^-^/CD45^-^/CD34^+^ VSELs and ~15% for the population of Lin^-^/CD45^-^/CD133^+^ VSELs. The right panel shows one representative set of results out of ten FACS analyses.

To exclude the possibility that EpoR has an indirect effect on VSEL expansion via accessory cells, we performed RT-PCR analysis of EpoR expression on FACS-purified murine and human VSELs and found that these cells express mRNAs for this receptor (Figure 2 panel A). Subsequently, we confirmed expression of EpoR on murine BM-purified VSELs by immunofluorescence staining (Figure 2 panel B). EpoR was also detected by FACS on ~15% of human CD34^+^ and CD133^+^ VSELs purified from human UCB (Figure 2 panel C).

### Functional EpoR is expressed by murine and human germline-derived cell lines

**Figure 3:**
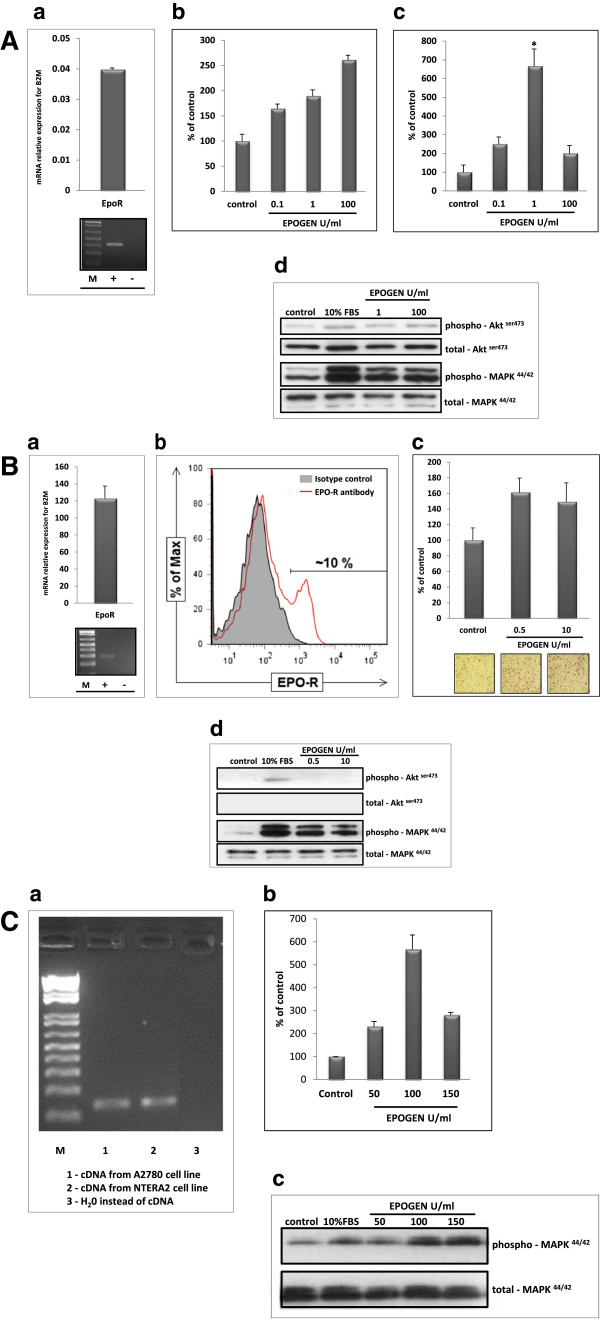
**Functional EpoR is expressed on murine and human germline-derived immortalized cell lines. Panel A. (a)** RT-PCR analysis of EpoR expression in the P19 murine teratocarcinoma cell line. Upper panel, RQ-PCR data, lower panel, SDS-PAGE gel of RT-PCR amplicon. The experiment was performed twice with similar results. **(b)**. The effect of erythropoietin (0–100 U/ml) on adhesion of P19 cells to fibronectin. Combined data from three independent experiments are shown. **(c)**. The effect of erythropoietin (0–100 U/ml) on the chemoattraction of P19 cells across Transwell membranes covered with gelatin. Combined data from three independent experiments are shown. **(d)**. Phosphorylation of AKT and p42/44 MAPK in P19 cells. A representative blot is shown. **Panel B. (a)** RT-PCR analysis of EpoR expression in the NTERA2 human teratocarcinoma cell line. Upper panel, RQ-PCR data; lower panel, SDS-PAGE gel of RT-PCR amplicon. Experiment was performed twice with similar results. **(b)**. Expression of EpoR on the NTERA2 cell line as measured by FACS. One representative histogram out of three experiments is shown. **(c)**. Effect of erythropoietin (0–10 U/ml) on adhesion of NTERA2 cells to fibronectin. Combined data from three independent experiments are shown. **(d)**. Phosphorylation of AKT and p42/44 MAPK in NTERA2 cells. One representative blot out of two is shown. **Panel C. (a)** RT-PCR analysis of EpoR expression in the A2780 human ovarian cancer cell line (lane 1). Lane 2, positive control (NTERA2 teratocarcinoma cell line). Lane 3, negative control (H_2_O instead of cDNA). The experiment was repeated twice with similar results. **(b)**. Effect of erythropoietin on chemoattraction of the A2780 ovarian cancer cell line across Transwell membranes. Combined data from two independent experiments are shown. **(c)**. Phosphorylation of p42/44 MAPK in the A2780 human ovarian cancer cell line stimulated by erythropoietin. A representative blot is shown.

Based on evidence that several cancer cell lines [11,25-27], including ovarian cancer cells [22,23,28,29], express EpoR, we became interested in whether functional EpoR is expressed by murine and human germline-derived cell lines. Figure 3 shows the results of our studies performed on the P19 murine teratocarcinoma cell line (panel A), the NTERA2 human teratocarcinoma cell line (panel B), and the A2780 human ovarian cancer cell line (panel C).Figure 3 panel A shows that murine teratocarcinoma cells express EpoR mRNA (a) and that this receptor is functional, as demonstrated by chemotaxis assay (b), adhesion assay (c), and direct signaling studies (d). Similarly, as shown in Figure 3 panel B, NTERA2 human teratocarcinoma cells express EpoR mRNA (a), EpoR is detectable by FACS on these cells (b), its functionality demonstrated by adhesion assay,(c) and direct signaling studies (d). Finally, in addition to our teratocarcinoma studies, which for the first time demonstrated EpoR expression by these cells, we also confirmed (Figure 3 panel C) that the human ovarian cancer cell line A2780 expresses EpoR mRNA (a) and that this receptor is functional according to chemotaxis assay (b) and direct cell signaling studies (c).

## Discussion

Our current understanding of stem cells in adult tissues suffers somewhat from the lack of a broader view of the stem cell compartment, which is a continuum between early stages of development and adult postnatal tissues. The recent hot debate on the existence in BM of early-development stem cells, such as spore-like stem cells, multipotent adult stem cells (MASCs) [30], multilineage-differentiating, stress-enduring (Muse) cells [31-33], multipotent adult progenitor cells (MAPCs) [34,35], marrow-isolated adult multilineage-inducible (MIAMI) cells [36], multipotent progenitor cells (MPCs) [30,37], and very small embryonic-like stem cells (VSELs) [12-14,38] with broader specification, resulted from the implicit challenge of these cells to the accepted hierarchy within the stem cell compartment in adult tissues.

We have proposed that VSELs initially identified by us [12,13,38] and confirmed by others [39-43] in murine and human adult tissues are a link between early stages of development and the compartment of adult stem cells that reside in adult tissues. The epigenetic mechanism identified in VSELs, which changes expression of certain genes involved in insulin/insulin-like growth factor signaling (IIS), keeps these cells in a highly quiescent state in adult tissues [44-47]. However, we have demonstrated that both murine and human VSELs may be specified into the hematopoietic lineage. This finding was indirectly corroborated by a recent report that the most primitive HSCs in murine BM, corresponding to long-term repopulating hematopoietic stem cells (LT-HSCs), like VSELs, are kept quiescent by changes in certain paternally imprinted genes involved in IIS [24,47]. In addition, in an old report [17,48] small murine BM-derived cells that share many characteristics with VSELs have been demonstrated to be a population of LT-HSCs.

The existence in BM of developmentally primitive stem cells with broader specification challenged the accepted hierarchy within the stem cell compartment in murine BM and the developmental position in this hierarchy of HSCs. Evidence has accumulated that HSCs can become specified from a population of migrating primordial germ cells (PGCs) during embryogenesis [3-5]. In support of this intriguing possibility, HSCs and PGCs are highly migratory cells, and specification of the first primitive HSCs in yolk sac blood islands as well as the origin of definitive HSCs in the aorta–gonado–mesonephros (AGM) region are chronologically and anatomically correlated with the developmental migration of PGCs in extra- and intra-embryonic tissues [7]. Moreover, murine VSELs express several markers, such as *Stella, Prdm14, Fragilis, Blimp1, Nanos3,* and *Dnd1*, that are shared with migratory primordial germ cells PGCs [18,19]. Based on these data, we have postulated a potential developmental link between PGCs, VSELs, and HSCs [3,5,19], and, as reported here in this study, the presence of EpoR on VSELs purified from murine BM and human UCB lends further support to this hypothesis.

We are aware that expression of EpoR (detected by FACS) on human VSELs was low (~15%). This result, however, depends on the affinity of antibodies against EpoR as well as the number of copies of the receptor on the cell surface. Moreover, we have demonstrated that VSELs residing in adult BM are somewhat heterogeneous, being most likely (more or less) pre-committed to various lineages [17,39-43,49]. We cannot exclude the possibility that the cells that highly express EpoR as detected by FACS staining are committed to HSCs. On the other hand, as we have demonstrated, VSELs do not express classical erythroid lineage markers such as Terr119 and CD71, and if this hematopoietic pre-commitment occurs in BM-residing VSELs, it occurs at the level of LT- HSCs.

In further support of a link between hematopoiesis and the germline, it has been demonstrated that PGCs isolated from murine embryos are able to grow hematopoietic colonies in vitro [3]. Furthermore, the presence of early-development stem cells with germline markers in BM that are able to differentiate along the germline lineage has already been demonstrated by several investigators. For example, murine BM SSEA-1^+^ cells stimulated by bone morphogenic factor 4 may differentiate Oct-4^+^Stella^+^Mvh^+^ cells into gamete precursors [50]. Similarly, a population of Oct-4^+^Mvh^+^Dazl^+^Stella^+^ cells present in murine BM has been reported to affect the recurrence of oogenesis in mice sterilized by chemotherapy [51,52], and Oct-4^+^Mvh^+^Stella^+^ cells isolated as Stra8–GFP cells from BM of Stra8–GFP transgenic mice express several molecular markers of spermatogonial stem cells and spermatogonia [6]. Furthermore, Oct-4^+^SSEA-1/3/4^+^ cells isolated from chicken BM are able to give rise to functional sperm after injection into testes [53]. In addition to BM, stem cells with germline potential have also been isolated from newborn mouse skin and porcine skin as well as porcine adipose tissue [54].

The identification of early-development stem cells in adult tissues raises several questions, such as: i) Are these cells functional and do they play a role in tissue/organ rejuvenation? ii) Are they involved in regulating life span of the individual? iii) Are they involved in regeneration of damaged tissues? iv) If regulatory mechanisms fail, could these cells give rise to malignancies?

To address this last question, the concept that adult tissues contain developmentally primitive cells with embryonic features that can lead to tumors is, surprisingly, not so novel. During the 19th and early 20th centuries, it was proposed that cancer may develop in populations of cells that are left in a dormant state in developing organs during embryogenesis. This so-called “embryonic rest hypothesis of cancer origin” was proposed by Virchow (1855), Durante (1874), and Conheim (1875) [55-57]. According to these pathologists, adult tissues contain embryonic remnants that normally lie dormant but that can be activated to become cancerous. In agreement with these theories, Wright (1910) proposed a germinal cell origin of nephroblastoma, and Beard (1911) proposed that tumors arise from displaced and activated trophoblasts or germ cells [58,59]. However, the putative cells responsible for these effects were at that time neither clearly identified nor purified from adult tissues. As mentioned above, we propose that mutated VSELs could be the missing link in this hypothesis by being the origin of these malignancies.

There are several pieces of evidence supporting the embryonic rest hypothesis of cancer development and the potential involvement of VSELs. First, there is the existence of classical germline tumors seen in patients with seminomas, ovarian tumors, yolk sac tumors, mediastinal or brain germ cell tumors, teratomas, and teratocarcinomas [60,61]. Second, several types of cancer cells express cancer testis (C/T) antigens (~40 identified), which are encoded by genes that are normally expressed only in the human germline but are also often expressed unexpectedly in various non-gonadal tumor types (e.g., gastric, lung, liver, and bladder carcinomas) [62,63]. Third, in several types of malignancies, embryonic markers, such as chorionic gonadotropin (hCG) and/or carcinoembryonic antigen (CEA), are detected in patient plasma [64]. Finally, it is known that several solid tumors (e.g., gastric, lung, bladder, and oral mucosa carcinomas as well as germinal tumors) express, like VSELs, the embryonic/germline transcription factor Oct-4 [65,66]. However, we are aware that more studies are needed to shed light on the potential role of VSELs in tumorigenesis.

In conclusion, we postulate that some support for the embryonic rest hypothesis of cancer development is lent by described by us herein presence of EpoR in both VSELs and germline-derived cell lines. On the other hand, since EpoR has been described in established cell lines of several malignancies [8,11,22,23,25], though it may be barely detectable on cells isolated from primary tumors [67,68], we envision that it could be a novel marker of cancer stem cells, for example, in gonadal malignancies (e.g., ovarian cancer stem cells). This, however, requires further study. Finally, since it is known that EPO signals in erythroid cells via EpoR-EpoR homodimers and in nonerythroid cells may signal via EpoR-CD131 heterodimers [69], thus while this paper was in review we evaluated by FACS expression of CD131 on human germ-line derived cells (VSELs, NTERA2, Ovarian Cancer) and did not find expression of CD131 (not shown).

## Competing interests

The authors declare that they have no competing interests.

## Authors’ contribution

MS carried out the study, performed FACS analyses, APB participated in study, studied teratocarcinoma cell lines participated in preparation of manuscript, PG – performed studies on ovarian cancer cells, JR - participated in preparation of manuscript and data analysis, KM – participated in data analysis, SSK - participated in manuscript writing, material collection and data interpretation, MK – performed RT-PCR studies, participated in preparation of manuscript and data analysis, MZR designed and coordinated the study, wrote the manuscript and provided funding. All authors read and approved the final manuscript.
